# Does household access to improved water and sanitation in infancy and childhood predict better vocabulary test performance in Ethiopian, Indian, Peruvian and Vietnamese cohort studies?

**DOI:** 10.1136/bmjopen-2016-013201

**Published:** 2017-03-07

**Authors:** Kirk A Dearden, Alana T Brennan, Jere R Behrman, Whitney Schott, Benjamin T Crookston, Debbie L Humphries, Mary E Penny, Lia C H Fernald

**Affiliations:** 1 Research and Quality Assurance; IMA World Health/Tanzania, Dar es Salaam, Tanzania; 2 Department of Global Health, Boston University, Boston, Massachusetts, USA; 3 Health Economics and Epidemiology Research Office, Faculty of Health Sciences, Department of Internal Medicine, School of Clinical Medicine, University of the Witwatersrand, Johannesburg, South Africa; 4 Department of Epidemiology, Boston University, School of Public Health, Boston, Massachusetts, USA; 5 Economics and Sociology Departments and Population Studies Center, University of Pennsylvania, Philadelphia, Pennsylvania, USA; 6 Population Studies Center, University of Pennsylvania, Philadelphia, Pennsylvania, USA; 7 Department of Health Science, Brigham Young University, Salt Lake City, Utah, USA; 8 School of Public Health, Yale University, New Haven, Connecticut, USA; 9 Instituto de Investigación Nutricional, Lima, Peru; 10 Division of Community Health Sciences, School of Public Health, University of California at Berkeley, Berkeley, California, USA

**Keywords:** water, sanitation, cognition, lower and middle income countries, PPVT, child development

## Abstract

**Objective:**

Test associations between household water and sanitation (W&S) and children's concurrent and subsequent Peabody Picture Vocabulary Test (PPVT) scores.

**Design:**

Prospective cohort study.

**Setting:**

Ethiopia, India, Peru, Vietnam.

**Participants:**

7269 children.

**Primary outcome measures:**

PPVT scores at 5 and 8 years. Key exposure variables were related to W&S, and collected at 1, 5 and 8 years, including ‘improved’ water (eg, piped, public tap or standpipe) and ‘improved’ toilets (eg, collection, storage, treatment and recycling of human excreta).

**Results:**

Access to improved water at 1 year was associated with higher language scores at 5 years (3/4 unadjusted associations) and 8 years (4/4 unadjusted associations). Ethiopian children with access to improved water at 1 year had test scores that were 0.26 SD (95% CI 0.17 to 0.36) higher at 5 years than children without access. Access to improved water at 5 years was associated with higher concurrent PPVT scores (in 3/4 unadjusted associations), but not later scores (in 1/4 unadjusted associations). 5-year-old Peruvian children with access to improved water had better concurrent performance on the PPVT (0.44 SD, 95% CI 0.30 to 0.59) than children without access to improved water. Toilet access at 1 year was also associated with better PPVT scores at 5 years (3/4 unadjusted associations) and sometimes associated with test results at 8 years (2/4 unadjusted associations). Toilet access at 5 years was associated with concurrent PPVT scores (3/4 unadjusted associations). More than half of all associations in unadjusted models (water and toilets) persisted in adjusted models, particularly for toilets in India, Peru and Vietnam.

**Conclusions:**

Access to ‘improved’ water and toilets had independent associations with children's PPVT scores that often persisted with adjustment for covariates. Our findings suggest that effects of W&S may go beyond subacute and acute infections and physical growth to include children's language performance, a critical component of cognitive development.

**Strengths and limitations of this study**

- Strengths include:
- Multiple country contexts, consistent measures of receptive vocabulary as well as individuals’ access to water and sanitation across early childhood.
- Adequate sample size, low attrition and a cohort design.
- Limitations include:
- Lack of information on hygiene, actual use of water and toilets, and contamination and infection.
- Lack of information on other variables that contribute to children's language development.

## Introduction

Over 250 million children aged under 5 years in low-income and middle-income countries (LMICs) are at risk for not attaining their developmental potential because they are stunted and/or living in poverty.^1^ Long-term consequences of poor early child development (ECD) include later reduced cognitive performance, educational attainment, adult productivity and increased intergenerational poverty risks.^1–3^

ECD programmes can take many forms, including promotion of good health and nutrition, support for safe and stimulating environments, protection from risks such as violence or abandonment, parenting support and early learning experiences, media, preschools and community groups.^4^ Poverty is the key underlying cause of poor child development; children living in poverty are exposed to many negative influences, including poor physical environments, inadequate nutrition, parental stress and insufficient cognitive stimulation.^5^ Undernutrition can influence brain development directly by affecting brain structure and function, or indirectly via poor physical or motor development, in addition to other pathways.^6–8^ Exposure to multiple co-occurring risks most likely contributes to greater disparities in developmental trajectories among children with differential exposure.^9–12^ This paper focuses on associations between specific aspects of children's physical environments—access to improved water and sanitation (W&S)—and childhood development as measured by performance on a test of receptive language.

Access to better W&S may benefit child development and cognition directly, and also by improving nutrition and/or reducing infection. In older children, lack of W&S may also lead to reduced school attendance due to behaviour changes (eg, water carrying) or poor health status. The several studies that address W&S and cognition have identified positive effects of improved sanitation on child cognition: one observational study^13^ found that poor household and neighbourhood sanitation were negatively associated with cognitive performance at age 5 years in univariate but not multivariate analyses. Another study^14^ reported that, relative to children whose mothers did not have safe toilet access during pregnancy, children whose mothers did have access had higher scores on a series of cognitive and memory tests. However, evidence addressing the direct relationship between W&S and cognition or language is still limited.^6^ ^11^ ^13^ ^15–17^

The connection between W&S and cognition is hypothesised to go from W&S to improved nutrition and then to improved cognition, as linear growth in the first 2 years of life has robust positive associations with cognitive development in individual studies,^18^ as well as in a larger meta-analysis.^19^ While few rigorous studies have assessed associations between W&S and child nutritional status, most of those that do have reported that improved sanitation is associated with better growth.^20–23^ However, a recent systematic review^24^ concluded that W&S interventions (including provision of soap and improvement of water quality) may have only small benefits on height-for-age z-scores (HAZ) among children aged <5 years, and no discernible effects on underweight (low weight-for-age) or wasting (low weight-for-height), raising concerns about the relevance of potential effects on cognition from improved nutrition.

Links between W&S and diarrhoea^11^ ^12^ and helminth infection^25^ are well documented, with some studies also showing connections between diarrhoea and helminth infections and cognition. Studies from Brazil and Bangladesh show relationships between diarrhoea and educational outcomes including late school entry, semantic fluency deficits, delayed verbal learning^7^ ^16^ ^26^ and reduced cognitive functioning.^21^ However, a systematic review of randomised trials found insufficient evidence of associations between helminth infections and cognitive performance.^15^

Infections arising from poor W&S such as hookworm can result in iron deficiency,^27–29^ and several trials have demonstrated that deworming and W&S interventions can improve anaemia status.^27^ ^29^ ^30^ Iron deficiency has known associations with cognitive performance.^31–33^

Early development consists of critical periods during which children are vulnerable to exposures.^34^ Delays in children's development occur cumulatively and start as early as conception, which supports arguments for early investments.^35^ The impact of different nutrients on children's development depends on timing, dose and duration of deficiencies.^8^ ^36^ Parenting practices and home environments also influence child development and may either accentuate or attenuate the effects of poverty, which directly affects child outcomes.^37^ Thus, potential intervention effects can vary according to timing, exposures and environmental conditions.^38^ For these reasons, it is important to consider trajectories of child development across a spectrum of ages, not just any one age.^39^

**Figure 1:**
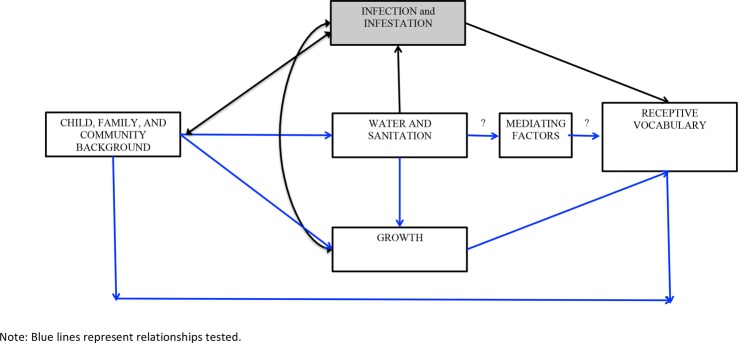
Conceptual framework for water and sanitation and receptive vocabulary.

This study's purpose is to investigate associations between living environments and, specifically, access to improved W&S, and performance on a test of receptive vocabulary ((the Peabody Picture Vocabulary Test (PPVT)) or the Spanish version of the test, Test de Vocabulario de Imagines Peabody (TVIP)) over early childhood.^i^ Figure 1 provides a conceptual framework linking water, sanitation and hygiene (WASH) and receptive vocabulary. Our hypotheses are:

- *H1*: Access to improved water is associated with higher PPVT scores, concurrently (H1A) and later in childhood (H1B).
- *H2*: Access to improved toilets is associated with higher PPVT scores, concurrently (H2A) and later in childhood (H2B).
- *H3*: Unadjusted associations are attenuated by adjusting for child, family and community characteristics.

## Materials and methods

### Study design and participants

We use the Young Lives (YL) Younger Cohort data on 8062 children in Ethiopia, India, Peru and Vietnam. Children were enrolled at 6–17 months (1 year), and followed at 4–5 years (5 years) and 7–8 years (8 years). The sampling strategy has been described previously (http://www.younglives.org.uk). YL teams used multistage designs to select 20 sentinel sites per country. While poor clusters were moderately oversampled, the final sample provided a diverse representation of social, geographic and demographic groups. The Indian sample covered only Andhra Pradesh and Telangana, while the other countries used nationwide samples. Within each cluster, ∼100 households with children aged 6–18 months were randomly selected. Children who moved were tracked and attrition was low. Poor rural children had higher mortality than wealthier urban children. Wealthier parents were more likely to refuse to participate and be untraceable.^40^ Still, results from probit analysis and Becketti, Gould, Lillard and Welch tests^41^ indicate limited evidence of attrition bias. We excluded children who were not aged 6–17.9 months at recruitment or who had changes in HAZ between rounds >4. Our final sample included 7269 children (Ethiopia 87.9% of observations, India 90.8%, Peru 90.1%, Vietnam 94.4%).

### Study indicators

*The PPVT* of receptive vocabulary was administered when children were 5 and 8 years (at 1 year, they were too young to test). Children were presented with sets of four pictures at a time and asked to select the picture that best represented a given word's meaning. Test items started with easy examples and became progressively harder. Children were not aware whether they made correct or incorrect choices. The PPVT in Ethiopia, India and Vietnam had 204 items; the one in Peru was based on the *TVIP*, and included 125 items. The PPVT and TVIP were adapted and standardised by YL researchers in each country and tested for reliability and validity.^42^

*Household access to improved W&S*: We used the WHO/Unicef Joint Monitoring Program (JMP) definition of ‘improved’ water source which includes piped water, public tap or standpipe, tubewell or borehole, protected dug well, protected spring, and rainwater collection. The JMP definition of ‘improved’ sanitation includes collection, storage, treatment, disposal, reuse and recycling of human excreta; drainage, disposal, recycling, and reuse of wastewater, storm water and household, industrial and hazardous solid waste.^43^ YL includes measures of household water and toilet access. We use ‘improved water’ and ‘improved toilets’, where ‘improved’ means that at a particular age children had access to improved water and toilets as per the JMP definition, not that access improved between data collection rounds. We do not know which children were consuming safe water, as improved water may be contaminated between sources and use.

*Other variables* included child's sex and age in months; asset index in the concurrent round;^44^ mother's height, age and schooling; father's schooling; household migration between rounds; and urban/rural residence at 1 year. Mother's height and parental schooling were unlikely to have changed between rounds. Urban/rural residence at 1 year was used instead of later residence under the assumption that the greatest impact of environment is when children are youngest. Community characteristics at 1 year included presence of hospitals and secondary schools, community population^ii^ and community wealth.^iii^ Online supplementary table S1 details all measures used along with information about when each was collected.

10.1136/bmjopen-2016-013201.supp1 supplementary tables

Informed consent was obtained from all participants. Online supplementary table S2 contains the STROBE statement for cohort studies and provides details about how our study addressed each criterion.

### Statistical analyses

We conducted ordinary least squares (OLS) unadjusted regressions with PPVT scores at 5 and 8 years as primary outcome measures. Our first models included the W&S indicators alone (improved water or improved toilets); the additional models adjusted for child, household, parent and community characteristics described above. We conducted analyses separately by country using SAS V.9.4 (Cary, North Carolina, USA).

We used the PROC MI SAS command for multiple imputation for missing values by the chained-equations method, which assumes that data were missing at random.^45^ All prediction equations included all covariates, namely child's sex and age in months; asset index in the concurrent round; mother's height, age and schooling; father's schooling; household migration between rounds; and urban/rural residence at 1 year. An indicator variable for country was added to the imputation procedure to inform the missingness and models were run separately for each country. PPVT values were not imputed. All models were fitted using 25 imputed data sets and estimated coefficients combined by averaging with the SAS MIANALYZE procedure. SEs were calculated using Rubin's rules for within-imputation and between-imputation SEs.^45^ All models were run clustered by community to take into account the correlation of error terms across observations.

## Results

At 5 years of age, PPVT raw scores (and SDs) were: Ethiopia 21.4 (13.4), India 27.3 (20.9), Peru 29.2 (17.8) and Vietnam 37.2 (18.1). At 8 years, raw scores were Ethiopia 68.4 (36.8), India 49.1 (26.7), Peru 46.8 (13.5) and Vietnam 77.2 (23.6). Raw scores should not be compared across countries because of differences in language of administration and number of test items (for Peru). Access to improved water was almost universal at age 8 years (89.1–97.0%) in all four countries (table 1). Vietnamese households had the least access to improved water at 1 year (9.7%), and the largest increase by 8 years (to 89.1%). Access to improved toilets was less universal: at 1 year, Ethiopia (21.4%) and India (25.3%) had the poorest toilet access; by 8 years, access more than doubled in Ethiopia (57.3%), but increased only slightly in India (35.0%).

**Table 1:** Descriptive statistics

	Ethiopia (n=1792)			India (n=1845)			Peru (n=1852)			Vietnam (n=1842)		
	Mean/per cent	SD	Number missing*	Mean/per cent	SD	Number missing*	Mean/per cent	SD	Number missing*	Mean/per cent	SD	Number missing*
Standardised PPVT scores												
5 years	−0.02	1.0	54	−0.01	1.0	120	0.01	1.0	68	0.01	1.0	211
8 years	−0.01	1.0	26	−0.00	1.0	39	0.02	1.0	113	0.01	1.0	114
Household-level water and sanitation												
Improved water 1 year	53.0		0	76.1		0	80.7		0	9.7		0
Improved water 5 years	82.3		0	95.0		0	88.0		0	83.7		0
Improved water 8 years	89.1		0	97.0		0	92.2		0	89.1		0
Improved sanitation 1 year	21.4		0	25.3		0	77.5		0	49.0		0
Improved sanitation 5 years	41.2		0	32.5		0	85.2		0	54.9		0
Improved sanitation 8 years	57.3		0	35.0		0	91.2		0	61.9		0
Child and household characteristics												
Age in months at 1 year	11.7	3.6	0	11.7	3.4	0	11.5	3.5	0	11.8	3.1	0
Age in months at 5 years	61.9	3.8	0	64.2	3.8	0	63.5	4.7	0	63.1	3.5	0
Age in months at 8 years	97.0	4.0	0	95.4	3.9	0	95.0	3.6	0	96.5	3.5	0
Child is female	47.0		0	46.2		0	49.8		0	48.8		0
Average consumption (5 and 8 years)	0.6	0.4	0	0.6	0.3	167	1.9	1.6	1	0.8	0.9	3
Mother's height	158.7	6.2	145	151.5	6.0	33	150.0	5.5	87	152.2	5.9	19
Mother’s completed schooling	3.0	3.8	14	3.7	4.4	4	7.8	4.4	15	7.0	4.0	15
Father's completed schooling	4.8	4.3	76	5.6	5.0	3	9.1	3.9	58	7.7	4.0	48
Mother’s age	27.5	6.4	47	23.6	4.3	8	26.8	6.8	12	27.1	5.7	3
Urban residence	35.0		0	24.9		0	66.1		0	19.4		0
Moved between ages 1 and 5 years	13.7		0	9.7		0	47.6		0	15.4		0
Moved between ages 5 and 8 years	9.3		0	2.4		0	77.4		0	0.2		0
Other community characteristics												
Community wealth	−0.03	2.7	0	−0.01	2.3	1	0.02	2.6	1	0.03	2.5	0
Community has a hospital	30.2		89	46.2		43	34.4		0	89.8		0
Community has a secondary school	34.5		89	43.7		43	78.1		0	98.2		0
Community population	8046.3	7261.0	89	3074.1	3660.0	150	4276.5	3958.9	198	10326.6	5812.6	0

*This column reflects the number of missing (and thus imputed) observations per variable.

PPVT, Peabody Picture Vocabulary Test.

*Hypothesis 1* (access to improved drinking water is associated with higher PPVT scores, concurrently and subsequently): Concurrent access to improved drinking water was frequently associated with cognition at 5 years (3/4 unadjusted associations), but mostly not when children were 8 years (1/4 unadjusted associations; table 2). Access to improved drinking water at 1 year was associated with cognitive performance at 5 years (3/4 unadjusted associations) and 8 years (all unadjusted associations). Also, drinking water at 5 years was associated with cognitive test scores at 8 years (3/4 unadjusted associations).

**Table 2:** Linear regression models for the association between improved water and PPVT test scores

n=1792	Unadjusted I	Child adjusted II	Child, household and parent adjusted III	Child, household, parent and community adjusted IV
*PPVT age 5*				
Improved water age 1				
Ethiopia	0.26**	0.14**	0.02	−0.08
	(0.17 to 0.36)	(0.04 to 0.24)	(−0.07 to 0.11)	(−0.17 to 0.02)
India	0.09	0.08	0.10	0.03
	(−0.02 to 0.20)	(−0.02 to 0.18)	(−0.01 to 0.19)	(−0.07 to 0.14)
Peru	0.31**	0.13*	−0.00	0.03
	(0.19 to 0.43)	(0.01 to 0.25)	(−0.11 to 0.11)	(−0.08 to 0.13)
Vietnam	1.02**	0.69**	0.37*	0.01
	(0.87 to 1.17)	(0.54 to 0.84)	(0.21 to 0.53)	(−0.20 to 0.21)
Improved water age 5				
Ethiopia	0.32**	0.32**	0.21**	0.11
	(0.20 to 0.43)	(0.20 to 0.43)	(0.10 to 0.31)	(−0.00 to 0.22)
India	0.01	−0.20*	0.23*	−0.14
	(−0.19 to 0.22)	(−0.40 to −0.01)	(−0.42 to −0.04)	(−0.34 to 0.06)
Peru	0.44**	0.26**	0.12	0.06
	(0.30 to 0.59)	(0.12 to 0.40)	(−0.00 to 0.25)	(−0.07 to 0.18)
Vietnam	0.16*	−0.06	−0.13*	−0.15*
	(0.04 to 0.28)	(−0.18 to 0.07)	(−0.25 to −0.01)	(−0.28 to −0.02)
*PPVT age 8*				
Improved water age 1				
Ethiopia	0.58**	0.35**	0.25**	0.09*
	(0.49 to 0.67)	(0.25 to 0.44)	(0.16 to 0.34)	(0.00 to 0.18)
India	0.14**	0.09	0.10*	0.04
	(0.03 to 0.25)	(−0.01 to 0.20)	(0.00 to 0.20)	(−0.06 to 0.15)
Peru	0.31**	0.13*	0.02	−0.00
	(0.20 to 0.43)	(0.02 to 0.24)	(−0.08 to 0.12)	(−0.10 to 0.10)
Vietnam	0.86**	0.56**	0.28**	0.04
	(0.71 to 1.02)	(0.41 to 0.72)	(0.12 to 0.45)	(−0.17 to 0.24)
Improved water age 5				
Ethiopia	0.13*	0.12	0.05	−0.07
	(0.01 to 0.29)	(−0.00 to 0.24)	(−0.07 to 0.16)	(−0.17 to 0.04)
India	0.22	0.08	0.05	−0.03
	(−0.01 to 0.44)	(−0.14 to 0.29)	(−0.17 to 0.26)	(−0.24 to 0.19)
Peru	0.42**	0.21**	0.10	0.07
	(0.26 to 0.57)	(0.07 to 0.35)	(−0.03 to 0.23)	(−0.06 to 0.20)
Vietnam	0.28**	0.06	0.04	−0.04
	(0.15 to 0.41)	(−0.08 to 0.19)	(−0.09 to 0.16)	(−0.17 to 0.09)
Improved water age 8				
Ethiopia	0.11	0.21**	0.05	−0.00
	(−0.04 to 0.26)	(0.07 to 0.35)	(−0.08 to 0.19)	(−0.13 to 0.12)
India	0.23	0.17	0.15	0.11
	(−0.06 to 0.51)	(−0.11 to 0.44)	(−0.12 to 0.42)	(−0.16 to 0.38)
Peru	0.36**	0.23**	0.17*	0.17*
	(0.18 to 0.54)	(0.07 to 0.40)	(0.02 to 0.32)	(0.02 to 0.32)
Vietnam	0.14	−0.09	−0.16*	−0.23**
	(−0.02 to 0.29)	(−0.24 to 0.06)	(−0.30 to −0.01)	(−0.38 to −0.07)

*p<0.05, **p<0.01. All adjusted models include both improved water and improved toilets. Child variables include age in months at outcome, sex and language the PPVT test was administered in. Household variables are asset index and household moved between rounds when there is more than one round of data on household toilet and water. Parent variables are age and height of mother, years of schooling of mother, and years of schooling of father. Community variables are urban residence, community population, community wealth, presence of a community hospital, and community has a public secondary school.

PPVT, Peabody Picture Vocabulary Test.

Country-specific results suggest that, with the exception of concurrent access to improved water and test scores at 8 years, all associations between access to improved water and test scores in Ethiopia, Peru and Vietnam were significant. For example, in Peru, 5-year-old children who had access to improved water had better concurrent performance on the PPVT (0.44 SD, 95% CI 0.30 to 0.59; unadjusted associations). Improved water was associated with higher PPVT scores at 5 years for Ethiopian children with access to improved water at 1 year (0.26 SD, 95% CI 0.17 to 0.36; unadjusted associations). In Vietnam, 8-year-old children who had access to improved water at 5 years had better performance on the PPVT (0.28 SD, 95% CI 0.15 to 0.41; unadjusted associations).

*Hypothesis 2* (access to improved toilets is associated with higher PPVT scores, concurrently and subsequently): Toilet access at 5 years was frequently associated with cognition at 5 years (3/4 unadjusted associations; table 3). Toilet access at 8 years was sometimes associated with cognitive test scores at 8 years (2/4 unadjusted associations).

**Table 3:** Linear regression models for the association between improved toilets and PPVT test scores

	Unadjusted	Child adjusted	Child, household and parent adjusted	Child, household, parent and community adjusted
n=1792	I	II	III	IV
*PPVT age 5*				
Improved toilets age 1				
Ethiopia	0.29**	0.24**	−0.04	−0.12*
	(0.18 to 0.40)	(0.13 to 0.35)	(−0.15 to 0.06)	(−0.23 to −0.01)
India	0.44**	0.48**	0.27**	0.40**
	(0.30 to 0.57)	(0.34 to 0.61)	(0.14 to 0.41)	(0.09 to 0.23)
Peru	0.48**	0.33**	0.21**	0.17**
	(0.36 to 0.59)	(0.22 to 0.45)	(0.10 to 0.31)	(0.07 to 0.27)
Vietnam	0.39**	0.31**	0.20**	0.08
	(0.29 to 0.50)	(0.20 to 0.41)	(0.10 to 0.31)	(−0.03 to 0.19)
Improved toilets age 5				
Ethiopia	0.04	0.01	−0.06	0.01
	(−0.05 to 0.14)	(−0.09 to 0.11)	(−0.15 to 0.03)	(−0.08 to 0.11)
India	0.33**	0.33**	0.08	0.09
	(0.21 to 0.46)	(0.21 to 0.46)	(−0.05 to 0.21)	(−0.05 to 022)
Peru	0.40**	0.30**	0.14*	0.09
	(0.26 to 0.53)	(0.17 to 0.43)	(0.02 to 0.25)	(−0.03 to 0.20)
Vietnam	0.42**	0.35**	0.19**	0.08
	(0.31 to 0.53)	(0.24 to 0.45)	(0.08 to 0.30)	(−0.04 to 0.19)
*PPVT age 8*				
Improved toilets age 1				
Ethiopia	0.60**	0.39**	0.13*	0.01
	(0.48. 0.71)	(0.29 to 0.50)	(0.02 to 0.23)	(−0.09 to 0.11)
India	−0.01	0.01	−0.09	0.04
	(−0.15 to 0.13)	(−0.14 to 0.15)	(−0.23 to 0.05)	(−0.13 to 0.21)
Peru	0.46**	0.23**	0.12*	0.08
	(0.35 to 0.58)	(0.12 to 0.34)	(0.02 to 0.22)	(−0.02 to 0.19)
Vietnam	0.34**	0.26**	0.17**	0.13**
	(0.23 to 0.45)	(0.15 to 0.37)	(0.06 to 0.27)	(0.02 to 0.24)
Improved toilets age 5				
Ethiopia	−0.05	−0.02	−0.08	0.04
	(−0.15 to 0.04)	(-0.11 to 0.07)	(−0.16 to 0.01)	(−0.05 to 0.12)
India	0.27**	0.28**	0.16*	0.20**
	(0.12 to 0.41)	(0.14 to 0.43)	(0.02 to 0.30)	(0.05 to 0.34)
Peru	0.41**	0.30**	0.16**	0.12*
	(0.28 to 0.55)	(0.17 to 0.42)	(0.05 to 0.28)	(0.04 to 0.24)
Vietnam	0.30**	0.25**	0.17**	0.17**
	(0.18 to 0.42)	(0.13 to 0.37)	(0.05 to 0.28)	(0.05 to 0.29)
Improved toilets age 8				
Ethiopia	−0.30**	−0.06	−0.07	0.01
	(−0.39 to -0.20)	(−0.15 to 0.03)	(−0.16 to 0.01)	(−0.08 to 0.09)
India	0.38**	0.37**	0.24**	0.26**
	(0.25 to 0.51)	(0.24 to 0.50)	(0.11 to 0.37)	(0.13 to 0.39)
Peru	0.13	0.11	0.09	0.08
	(−0.03 to 0.29)	(−0.15 to 0.01)	(−0.05 to 0.23)	(−0.06 to 0.22)
Vietnam	0.28**	0.25**	0.15*	0.11*
	(0.17 to 0.40)	(0.13 to 0.36)	(0.03 to 0.26)	(−0.00 to 0.22)

*p<0.05, **p<0.01. All adjusted models include both improved water and improved toilets. Child variables include age in months at outcome, sex and language the PPVT test was administered in. Household variables are asset index and household moved between rounds when there is more than one round of data on household toilet and water. Parent variables are age and height of mother, years of schooling of mother, and years of schooling of father. Community variables are urban residence, community population, community wealth, presence of a community hospital, and community has a public secondary school.

PPVT, Peabody Picture Vocabulary Test.

Toilet access at 1 year was always associated with cognitive test scores at 5 years (4/4 unadjusted associations) and often associated with test results at 8 years of age (3/4 unadjusted associations). Likewise, three of four unadjusted associations between toilet access at 5 years and cognitive test scores at 8 years were significant.

Country-specific results suggest that toilet access was associated with cognitive test scores at all ages in Vietnam and all but at 8 years in Peru, in unadjusted models. With the exception of toilet access at 1 year and test scores at 8 years, all unadjusted associations in India were likewise significant.

*Hypothesis 3* (unadjusted associations are attenuated by adjusting for child, family and community characteristics): Adjusting for covariates almost always attenuated associations among water and toilet access and test scores. Of 14 associations found to be significant in unadjusted models, 79% (11) of associations persisted after adjusting for child covariates (child age, sex and language used for the PPVT), 43% (6) remained after adjusting for child, household and parent characteristics, and 14% (2) persisted after adjusting for community characteristics as well.

While associations between access to improved toilets and PPVT scores were attenuated somewhat after adjusting for covariates, many remained significant. Of the 15 associations found to be significant in unadjusted models, 100% (15) of associations persisted after adjusting for child covariates, 87% (13) remained after adding household covariates and 53% (8) persisted once community characteristics were included in models.

In some cases, associations were significant, but in unexpected directions. In adjusted models for Vietnam, access to improved water at age 8 years was negatively associated with PPVT scores at age 8 years. In Ethiopia, in the fully adjusted model, access to improved toilets at 1 year was inversely associated with PPVT scores at 5 years. We are not certain why in some cases toilets and PPVT scores were inversely related; however, it may be that (1) household variables proxy for community characteristics in unadjusted estimates, (2) multiple community measures weaken the observed relationship with household measures, and (3) the way toilets are used may explain these differences. For example, a 1-year-old most likely uses nappies or rags, whereas older children do not. Studying potential pathways between improved W&S and PPVT scores could provide explanations for these apparent anomalies.

We conducted tests of heterogeneity to determine if there were statistically significant differences in coefficients, by countries. There were few differences and in cases where coefficients differed by country, there was no discernible pattern in these differences (no single country was consistently different from the others (results not presented in table form but available from the lead author)).

## Discussion

We examined improved W&S access to determine if these dimensions of children's physical environment were associated with performance on tests of cognition. Using longitudinal data from four LMICs, we found that concurrent access to improved water was associated with higher receptive language scores at 5 years. Access to improved water at 1 year was associated with language performance at 5 and 8 years and access to improved water at 5 years was associated with language test scores at 8 years. Toilet access at 5 years was frequently associated with cognition at 5 years in unadjusted models. Toilet access at 1 year and 5 years was significantly associated with cognitive test scores at 5 years and 8 years in unadjusted models. Toilet access was associated with cognitive test scores in India, Vietnam, and Peru in unadjusted models. Adjusting for covariates attenuated associations found in unadjusted models but many associations persisted, suggesting that while child, household, parent and community characteristics were associated with performance on the PPVT, access to improved water and toilets may have an independent relationship to children's scores on cognitive tests, both concurrently and over a 7-year period.

Our observational study has several strengths including multiple country contexts, adequate sample size, low study attrition and a cohort design with consistent measures of individual access to W&S and vocabulary test data across early childhood. Limitations include lack of information on hygiene, actual use of water and toilets, and contamination and infection. Furthermore, we lack information on other variables that may contribute to children's language development, such as psychosocial stimulation, exposure to toys and books in the home, and engagement of caregivers in processes of connecting with and educating their children. Future research would benefit from including this information. Our findings are not necessarily generalisable, but do include four LMICs on three continents and therefore make an important contribution to understanding associations between improved water, improved sanitation and performance on a test of cognition.

Our findings are similar to the two studies that we identified that explicitly examine associations between lack of improved W&S access and children's performance on tests of cognition. Similar to our findings, Santos *et al*,^13^ studying a Brazilian cohort, reported negative associations between inadequate sanitary conditions (including household water supply and sewage disposal) when children were <3 years and the Wechsler Pre-School and Primary Scale of Intelligence scores at 5 years. Their analytic approach, which was similar to ours, adjusted for child covariates, the physical and psychosocial environment, and socioeconomic status. They found that household and community sanitation, coupled with psychosocial stimulation, accounted for an additional 6.3% of variation in cognitive test scores beyond the variation explained by socioeconomic status alone. The same study reported that poor sanitation and poor psychosocial stimulation appeared to act causally on cognitive performance and did not seem to be mediated by infection or nutrition.

For Indonesia, Wulan *et al*^14^ assessed associations between household safe water and toilet access during the prenatal period and childhood cognition 9–12 years later. They found that relative to children whose mothers did not have safe toilet access during pregnancy, children whose mothers had had access scored higher on the Digit Span Forward, Information, Block Design, and Word List Memory tests, after adjusting for several covariates, including socioeconomic status and home environment.

While the impacts of some risk factors on child development are known, whether and how children's physical environments affect developmental outcomes remains largely unstudied. Additionally, little is known about periods of greatest vulnerability to environmental insults, which could inform timing and composition of interventions. In many LMICs, faecal contamination of home environments is nearly universal and, as Ngure *et al*^10^ note, such exposure may contribute to a constant and cumulative health risk precisely when children should be experiencing their greatest growth. Risks such as faecal contamination, malnutrition and lack of parental cognitive stimulation are likely to interact. For example, anaemic infants may be reluctant to freely explore their surroundings and may be more likely to cling to caregivers; parents who perceive their children to be small for their age may provide less age-appropriate stimulation.^46^ ^47^ Additionally, parents may be less likely to interact with their children if they are frequently apathetic because of sickness.^10^

Our findings reinforce the possibility that the impacts of W&S may go beyond subacute and acute infections and physical growth to include children's cognitive performance. On the basis of these results, we think that rigorous longitudinal investigations should add specific indicators designed to determine the concurrent and long-term impact of W&S on cognition and other child development outcomes. Such studies would benefit from an investigation of both the mechanisms and multiple pathways to enhanced cognition, including enhanced parental engagement and responsiveness; greater access to toys, books and learning materials; reduced infection; and growth. Consideration should be given to how different caregiving practices such as infant feeding, play, stimulation and other behaviours mediate these pathways. A better understanding of the role of certain caregiving practices could suggest specific interventions that can then be tested in trials. It may be that the same factors that influence exposure to unhygienic conditions also contribute to cognitive development. For example, attentive mothers who proactively keep household compounds clean may also be more likely to stimulate their children's development than mothers who do not keep the household clean. Currently, there are two major randomised intervention studies examining the potential role of environmental enteropathy in childhood anaemia, stunting and morbidity (SHINE in Zimbabwe; WASH Benefits in Bangladesh and Kenya),^48^ ^49^ as well as a large prospective, longitudinal study (MAL-ED) in Bangladesh, Brazil, India, Nepal, Pakistan, Peru, South Africa and Tanzania.^50^ The results of these trials may provide some clarity on the possible physiological mechanisms between environmental influences and child growth and development.

Non-governmental organisations, Unicef and the WHO promote integrated early childhood development interventions. Programmes that focus on young children's broader physical environments as well as opportunities for psychosocial stimulation need to be developed and tested for impact. As a starting point, WASH programmes could integrate critical messages on early childhood development and ECD interventions could include more focus on WASH.

## Footnotes

i Information on hygiene is not available for all countries; consequently, we do not explore associations between hygiene and children's language scores.

ii Community data were reported by community leaders.

iii Community wealth is the average asset index for the other households in the same community.
