# Supplementary material for: Does household access to improved water and sanitation in infancy and childhood predict better vocabulary test performance in Ethiopian, Indian, Peruvian and Vietnamese cohort studies?
Source: BMJ Open. 2017 Mar 7;7(3):e013201. doi: 10.1136/bmjopen-2016-013201 (PMC5353302; doi:10.1136/bmjopen-2016-013201)
Supplement: supplementary tables [file bmjopen-2016-013201supp_tables.pdf]

Supplemental tables

Table S1. Timing of measurement of outcomes, exposures, and other covariates

Table S2. STROBE Statement for cohort studies

Supplemental table S1. **Timing of measurement of outcomes, exposures, and other covariates**

	**Round 1 y (1 y old)**	**Round 2 (5 y old)**	**Round 3 (8 y old)**
**Outcomes** (child level)			
PPVT		X	X
**Exposure** (household level)			
Improved water	X	X	X
Improved toilets	X	X	X
**Exposure** (community level)*			
Open defecation	X		
% in community w/ improved toilet facilities	X		
% in community w/ improved water	X		
Animal excrement in street		X	
Animal feces at water sources		X	
Garbage dumped at water sources		X	
Sewerage available in community		X	
**Other covariates**			
Child's age in months	X	X	X
Child's sex	X		
Average household consumption		X	
Mother's height	X		
Mother's completed schooling		X	
Father's completed schooling		X	
Mother's age	X		
Urban residence	X		
Moved between ages 1 y and 5 y		X	
Moved between ages 5 y and 8 y			X
Community wealth	X		
Community has hospital	X		
Community has secondary school	X		
Community population	X		

* All community level exposure variables are linked to the community in which the child lived at age 1 y, the time when exposure is likely most critical. However, this link requires the assumption that these exposure variables remain stable from round 1 to round 2 in a given community.

Supplemental table S2. **STROBE Statement for cohort studies**

	Item No	Recommendation	
**Title and abstract**	1	(*a*) Indicate the study's design with a commonly used term in the title or the abstract	The study examined four cohorts of children.
		(*b*) Provide in the abstract an informative and balanced summary of what was done and what was found	The outcome measure (Peabody Picture Vocabulary Test or PPVT score) and the statistical analyses performed to test associations between PPVT scores and measures of water and sanitation (W&S) are described in the abstract. Results are presented for unadjusted and adjusted models.
**Introduction**			
Background/rationale	2	Explain the scientific background and rationale for the investigation being reported	The Background explains the determinants and consequences of poor child development and the literature on associations between W&S and cognition, W&S and nutrition, and W&S and infectious diseases, including diarrhea and helminth infections.
Objectives	3	State specific objectives, including any pre-specified hypotheses	The article's purpose was to investigate associations between access to improved W&S and performance on a test of receptive vocabulary. The Introduction also outlines the three hypotheses used to test these associations.
**Methods**			
Study design	4	Present key elements of study design early in the paper	The cohorts studied, sampling, how study measures were assessed, and statistical analyses are presented immediately after the introduction.
Setting	5	Describe the setting, locations, and relevant dates, including periods of recruitment, exposure, follow-up, and data collection	The four country cohorts are described, along with children's age at study enrolment and follow-up. This was an observational study; there were no exposures to report.
Participants	6	(*a*) Give the eligibility criteria, and the sources and methods of selection of participants. Describe methods of follow-up	The article indicates eligibility criteria (child 6-17.9m at recruitment, changes in HAZ between rounds less than 4). The article also describes how children were sampled (multi-stage sampling with sentinel sites and randomly selected age eligible households within clusters). Additional details about methodology, including follow-up methods, are referenced in numerous other studies and are available from the Young Lives website (http://www.younglives.org.uk).
		(*b*) For matched studies, give matching criteria and number of exposed and unexposed	Not applicable.
Variables	7	Clearly define all outcomes, exposures, predictors, potential confounders, and effect modifiers. Give diagnostic criteria, if applicable	The primary outcome of interest (children's PPVT scores), predictors (measures of W&S), and potential confounders are described in the article.
Data sources/ measurement	8*	For each variable of interest, give sources of data and details of methods of assessment (measurement). Describe comparability of assessment methods if there is more than one group	Methods of assessment are described in detail for both the outcome of interest and for predictor variables.
Bias	9	Describe any efforts to address potential sources of bias	The article references the work others have done to examine potential bias, including attrition (limited evidence for bias).
Study size	10	Explain how the study size was arrived at	Sampling procedures are described in the article. Details about sample size calculations are available from the Young Lives website (http://www.younglives.org.uk).
Quantitative variables	11	Explain how quantitative variables were handled in	Supplemental table S1 details all measures used, along with information about when each was collected. The article

		the analyses. If applicable, describe which groupings were chosen and why	also provides the rationale for selecting data from specific rounds.
Statistical methods	12	(*a*) Describe all statistical methods, including those used to control for confounding	Analyses included OLS unadjusted regressions with primary outcome measures (PPVT scores at 5y and 8y). Model construction and how potential confounders were handled is also described.
		(*b*) Describe any methods used to examine subgroups and interactions	Data were analysed by country.
		(*c*) Explain how missing data were addressed	The article indicates procedures for multiple imputation for missing values (chained equations method) and the inclusion of all covariates for prediction equations.
		(*d*) If applicable, explain how loss to follow-up was addressed	Children who moved between rounds were tracked by collecting detailed contact information at each round. All efforts were made to track children within their country of origin, but children who migrated to another country were not tracked.
		(*e*) Describe any sensitivity analyses	We did not conduct sensitivity analyses.
**Results**			
Participants	13*	(a) Report numbers of individuals at each stage of study—eg numbers potentially eligible, examined for eligibility, confirmed eligible, included in the study, completing follow-up, and analysed	The article describes initial and final sample sizes and gives attrition rates.
		(b) Give reasons for non-participation at each stage	Poor, rural children had higher mortality than wealthier, urban children. Wealthier parents were more likely to refuse to participate and be untraceable.
		(c) Consider use of a flow diagram	Not included but available at the Young Lives website.
Descriptive data	14*	(a) Give characteristics of study participants (eg demographic, clinical, social) and information on exposures and potential confounders	Table 1 includes information on children's age, sex, mother's height, mother's and father's schooling, residence, migration, household consumption, and a number of community characteristics.
		(b) Indicate number of participants with missing data for each variable of interest	Missing data were imputed for W&S and for confounders. Data for the outcome of interest (PPVT) were not imputed. The final sample sizes for each country indicate how many children provided PPVT scores.
		(c) Summarise follow-up time (eg, average and total amount)	Length of time between data collection rounds is provided.
Outcome data	15*	Report numbers of outcome events or summary measures over time	Outcome events included PPVT scores at ages 5y and 8y.
Main results	16	(*a*) Give unadjusted estimates and, if applicable, confounder-adjusted estimates and their precision (eg, 95% confidence interval). Make clear which confounders were adjusted for and why they were included	Results for unadjusted and adjusted models are presented, both in the body of the article and in the tables. The significance of estimates is also presented. Models included confounders at the child level; child, household, and parent level; and child, household, parent, and community level.
		(*b*) Report category boundaries when continuous variables were categorized	Most continuous variables (including PPVT scores, age, maternal height, and community wealth were not categorized). The exception was maternal and paternal education (in years completed) which was classified in a standard fashion (did not complete primary school, completed primary school, etc.).
		(*c*) If relevant, consider translating estimates of	The article presents coefficients for linear regression models, along with associations.

		relative risk into absolute risk for a meaningful time period	
Other analyses	17	Report other analyses done—eg analyses of subgroups and interactions, and sensitivity analyses	Analyses were stratified by country. Tests of heterogeneity were conducted to determine if there were statistically significant differences in coefficients, by country. Analyses included testing of interaction terms, specifically, access to improved water and access to improved toilets, interacted separately when children were 1y, 5y, and 8y old.
**Discussion**			
Key results	18	Summarise key results with reference to study objectives	Findings are presented by hypothesis.
Limitations	19	Discuss limitations of the study, taking into account sources of potential bias or imprecision. Discuss both direction and magnitude of any potential bias	Limitations are acknowledged and include lack of information on hygiene, actual use of water and toilets, and contamination and infection. There are other factors that contribute to children's language development and these are not captured in the present study.
Interpretation	20	Give a cautious overall interpretation of results considering objectives, limitations, multiplicity of analyses, results from similar studies, and other relevant evidence	Adjusting for covariates attenuated results. Comparisons are made to results from similar studies. Limitations of the study are described in detail and findings are presented cautiously, acknowledging such limitations. The article also spells out additional research needed, including research that addresses the limitations of the present study, as well as others' research.
Generalisability	21	Discuss the generalisability (external validity) of the study results	Study results are not necessarily generalizable but do include four low- and middle-income countries on three continents and therefore make an important contribution to the literature.
**Other information**			
Funding	22	Give the source of funding and the role of the funders for the present study and, if applicable, for the original study on which the present article is based	This information is presented with the article.
